# Impact of percutaneous ventricular septal defect closure on left ventricular remodeling and function

**DOI:** 10.1186/s43044-021-00215-z

**Published:** 2021-10-12

**Authors:** Amr Abdel Aal, Housam M. Hassan, Dina Ezzeldin, Maiy El Sayed

**Affiliations:** Cardiology Department, Faculty of Medicine, Helwan University, Cairo, Egypt

**Keywords:** Ventricular septal defect, Left ventricle remodeling, Echocardiography, Percutaneous closure, Congenital heart defect

## Abstract

**Background:**

Ventricular septal defect (VSD) is the most common congenital heart disease. In patients with large VSD, left side chambers are subjected to volume overload with subsequent chambers dilatation and eccentric left ventricular hypertrophy. Percutaneous closure of VSD has been shown to be an effective method with equal safety and efficacy when compared to surgery. The effect of VSD closure on LV remodeling has been mainly assessed in patients treated with surgery and to date published data remain scarce. Therefore, we aim to evaluate the effect of percutaneous VSD closure on different LV parameters.

**Results:**

Seventeen patients (median age 6 years (IQR 4.75–8 years), 70.6% females) who underwent percutaneous VSD closure were enrolled in the study. Sixteen patients (94%) had perimembranous VSD, and one patient had muscular VSD. The procedure was successful in all patients with no major complications. Nit Occlud® Lê coil device was implanted in 16 patients (94%), and one patient received Amplatzer PDA duct occlude device. At 6-months follow-up, there was a significant reduction in indexed LV dimensions [LVEDD/BSA (median 46.5 mm/m^2^ vs. 42.9 mm/m^2^, *p* = 0.03), LVESD/BSA (median 31.7 mm/m^2^ vs. 26.7 mm/m^2^, *p* = 0.02)], indexed LV volumes [LVEDV/BSA (median 52.6 ml/m^2^ vs. 37.3 ml/m^2^, *p* = 0.02), LVESV/BSA (median 31.7 ml/m^2^ vs. 23.3 ml/m^2^, *p* = 0.02)] and indexed LV mass (median 62.4 gm/m^2^ vs. 57.9 ml/m^2^, *p* = 0.01). There was a significant reduction in LVEDD Z-score (*p* = 0.01) and LVESD Z-score (*p* = 0.04). There was no significant change in LV EF.

**Conclusions:**

Percutaneous VSD closure is associated with improvement of various LV parameters with consequential favorable LV remodeling and function.

## Background

Ventricular septal defect (VSD) is the most common form of congenital heart diseases, being responsible for 20% of the various congenital heart diseases with an overall incidence rate of 3–3.5 per 1000 live birth [1, 2]. VSD occurs in various anatomic locations along the interventricular septum; perimembranous, muscular, outlet and inlet locations with 70–80% of defects are in the perimembranous position [1].

In patients with large VSD associated with significant left to right shunt in the absence of pulmonary vascular disease, left side chambers are subjected to volume overload secondary to increased pulmonary blood flow and venous return. This will eventually lead to left side chambers dilatation and eccentric left ventricular hypertrophy due to amplification of left ventricular (LV) wall stress [3].

Surgical closure of VSD is the gold standard for treatment of large VSD. Percutaneous closure of VSD was shown to be an effective method with comparable success rate and major complications to surgical closure and with shorter hospital stay and better cosmetic outcome [4].

Longstanding volume overload with VSD may lead to irreversible structural changes at myocyte level. Early VSD closure assures reversibility of such changes and normalization of LV dimensions, volumes and mass which follow different pace [5, 6]. The effect of percutaneous closure of VSD on various LV parameters and its time course is not known, especially when associated with residual shunt and/or valvular regurgitation which may hinder complete removal of volume overload.

The aim of this study is to detect the effect of percutaneous VSD device closure on left ventricular remodeling and function.

## Methods

### Study population

From June 2015 to February 2017, patients diagnosed with VSD and have undergone percutaneous closure were recruited and followed-up for a period of 6 months post-procedure.

Inclusion criteria were: (1) VSD located within the muscular septum or in the perimembranous septum, (2) ≥ 8 kg of body weight, (3) pulmonary vascular resistance < 7 woods unit, (4) proximity of ventricular septal defect to Aortic valve ≥ 3 mm by TTE and (5) significant left to right shunt with pulmonary to systemic blood flow ratio (Qp/Qs) > 1.5, supported by the presence of (a) cardiomegaly on CXR (defined as cardiothoracic ratio > 0.5) and/or (b) clinical manifestations as recurrent respiratory infections and /or symptoms of heart failure. Exclusion criteria included: (1) VSD associated with the presence of other congenital cardiac abnormalities that require surgical treatment, (2) contraindication to antiplatelet therapy.

All patients were subjected to history taking, general and cardiac examination and growth assessment [weight, height, body surface area (BSA) and body mass index (BMI)]. Any clinical evidence of extra cardiac lesions detected during examination was recorded. A 12-lead electrocardiogram was performed before and immediately after the procedure and at 1- and 6-months post-VSD closure to detect any rhythm or conduction abnormalities.

A detailed and informed written consent was obtained from all the eligible patients or parents. The study was approved by the institutional review board and local ethical Committee.

### Echocardiographic study

Patients underwent transthoracic echocardiography using IE33 Phillips echocardiography machine equipped with a phased array transducer of suitable frequency. The echocardiography study was done before and immediately after the procedure and repeated at 1- and 6-months post-VSD closure. LV internal dimensions, septal and posterior wall thickness were measured at end-diastole and end-systole from cross-sectional guided M-mode echocardiograms. Measurements were done at the level of tips of mitral leaflets in parasternal long axis view and were averaged over 3 cardiac cycles. End-diastole was defined as the frame at which the mitral valve closes and end systole as the frame preceding mitral valve opening [7].

Pre-procedure echocardiography focused on full assessment of the VSD (site, size, relation to surrounding structures, proximity to the aortic valve and presence of any aneurysmal tissue) and assessment of various LV parameters (LV dimensions, LV volumes and LV mass using 2D and M-mode methods and ejection fraction (EF) using Biplane method). LV dimensions and volumes were indexed to the body surface area. Aneurysmal tissue was considered complete if it forms a complete circle around the defect; otherwise, it was considered incomplete. The left ventricular end-diastolic dimension Z-score (LVEDD-Z) and left ventricular end-systolic dimension Z-score (LVESD-Z) were calculated as the number of standard deviations from the mean value of the normal population relative to the BSA [8].

Doppler echocardiography was done to determine pressure gradients across VSD, pulmonary flow: systemic flow ratio (Qp:Qs), presence of aortic regurgitation and estimated pulmonary artery systolic pressure from tricuspid regurgitation velocity if detected by color flow mapping before VSD closure.

Post-percutaneous closure of the VSD, an echocardiography was done immediately to detect any complications related to the procedure and assess the result of procedure (device alignment with interventricular septum (IVS), encroachment and relation of the device to the surrounding structures, residual shunt across the device, Qp:Qs in the presence of residual shunt and presence of aortic regurgitation).

During the period of follow-up, an echocardiography was done at 1- and 6-months post-procedure to record changes in indexed LV parameters and detect the fate of any residual shunt present post-procedure.

### Percutaneous VSD closure

Percutaneous closure of VSD was performed under general anesthesia in all patients. All procedures were guided by trans-thoracic echocardiography or trans-esophageal echocardiography and fluoroscopy. All patients received intravenous heparin (100 units/kg) and antibiotics before the procedure. Right and left heart catheterization was preformed through percutaneous transfemoral route.

Assessment of the defect was performed in multiple views. The angiographic view that best profiled the defect was used as a reference for closure. In most cases, modified left anterior oblique 60° cranial 20° and left anterior oblique 45° cranial 45° were used. Measurements of the defect size from left and right ventricular sides and its distance from the aortic and tricuspid valve were confirmed using angiography and echocardiography. Device selection was done based on operator preference on case-by-case bases. Patients were prescribed antiplatelet therapy in the form of oral aspirin 5 mg/kg/day for 6 months.

### Definition of outcomes

#### Procedural success

Procedural success was defined by device implantation in the appropriate position with no need for surgery due to significant residual shunt and/or valve regurgitation [9].

#### Residual shunt

A residual shunt was diagnosed if the color-Doppler flow mapping showed a left to right shunt across the interventricular septum. It was classified as follows: trivial (< 1 mm color jet width), small (1–2 mm color jet width), moderate (> 2–4 mm color jet width) and large (> 4 mm color jet width) [9].

#### Statistical analysis

Statistical analysis was carried out using SPSS 20. Numerical data were presented as number and percentages, while categorical data were presented as median and interquartile range (IQR). Comparison between pre-closure and post-closure data was done using Wilcoxon Signed-Rank test. A probability value of *p* < 0.05 was considered statistically significant.

## Results

### Patient’s characteristics

This study included 17 patients who underwent percutaneous closure of VSD from June 2015 to February 2017 and completed follow-up period. The demographic characteristics of the patients are shown in Table 1. Median age of the patients was 6 years (IQR 4.75–8 years) and females represented two-thirds of the patients. None of the patients showed clinical evidence of extra cardiac lesions.

**Table 1:** Demographic characteristics of the patients

Parameters	Median (IQR)
Female [No. (%)]	12 (70.6%)
Age (year)	6 (4.75–8)
Weight (Kg)	22 (14–25.75)
Height (cm)	115 (100–126)
BSA (m^2^)	0.82 (0.6–0.91)
BMI (kg/m^2^)	18 (16.1–20.3)

BMI: Body mass index, BSA: body surface area, IQR: interquartile range

### Baseline echocardiographic data

VSDs and baseline echocardiographic characteristics are demonstrated in Tables 2 and 3, respectively. Ninety-four percent of the patients had a single VSD (one patient had two ventricular septal defects), and 94% had perimembranous ventricular septal defects (one patient had mid muscular VSD).

**Table 2:** VSDs characteristics

ID	Gender	Age (years)	Weight (Kg)	Height (Cm)	AV-VSD (mm)	VSD LV side size (mm)	VSD RV side size (mm)	Aneurysm	Multiple VSD jets	Qp/Qs
1	F	2.67	14	75	6	10	5	Yes	No	1.9
2	F	2.17	12	68	7	6	3	Yes	No	2.4
3	F	5.5	22	108	4.6	5	3.3	No	No	2.3
4	F	10.25	37	138	9	14	8.5	Yes	Yes	2.8
5	F	6	25	118	9	6	3	Yes	No	3.8
6	M	7	24	116	5	3	2	Yes	Yes	2.5
7	F	7	19	115	8	5	3	No	No	1.6
8	F	4.5	13	102	4	12.5	6.5	Yes	Yes	4
9	M	4	13	88	4	13	7.5	Yes	Yes	2
10	M	11	28	132	5	13	6	No	No	1.8
11	F	13	48	144	7	14	7	Yes	No	2
12	M	8	22	124	3	12	6	Yes	No	1.6
13	M	5	19	105	4.7	10	5	Yes	No	1.8
14	F	6	25	117	5	10	5.5	Yes	No	2
15	F	17	60	155	4	13	7	Yes	No	2.4
16	F	3.3	16	100	5	10	6	Yes	No	1.8
17	F	2.75	13	80	3	11	6	Yes	Yes	1.9

AV-VSD: Distance between VSD and rim of Aortic valve, F: female, M: male, Qp/Qs: ratio of pulmonary blood flow to systemic blood flow, VSD: ventricular septal defect

**Table 3:** Baseline echocardiographic characteristics

Measurements		Baseline [median (IQR)]
LVEDD (mm)		40 (34–42)
LVESD (mm)		24 (22–28)
LVEDV (ml)		41(32–50.75)
LVESV (ml)		25 (18–28)
LV mass (gm)		49.5 (37.5–65)
LVEDD/BSA(mm/m^2^)		46.51(45.45–52.56)
LVESD/BSA(mm/m^2^)		31.69 (27.91–35.82)
LVEDV/BSA(ml/m^2^)		52.56 (47.14–58.54)
LVESV/BSA(ml/m^2^)		31.7 (25.58–34.69)
LV mass/BSA (gm/m^2^)		62.4(55.2–78.85)
LVEDD Z-score		1.2 (0.0175–1.76)
LVESD Z-score		1.7 (0.59–2.49)
EF (%)		65 (60–69)
VSD-AV distance		5 (4–6.75)
VSD size LV (mm)		10 (6–13)
VSD size RV (mm)		6 (3.3–6.5)
Aneurysmal tissue [no (%)]	Incomplete	10 (58.8%)
	Complete	4 (23.6%)
	No	3 (17.6%)
Gradient across defect (mmHg)		85 (75–93)

AV: Aortic valve, BSA: body surface area, EF: ejection fraction, LA: left atrium, LV: left ventricular, LVEDD: left ventricular end diastolic dimension, LVESD: left ventricular end systolic dimension, LVEDV: left ventricular end diastolic volume, LVESV: left ventricular end systolic volume, RV: right ventricle, VSD: ventricular septal defect

### Percutaneous closure outcome

Nit Occlud® Lê VSD coil device (pfm medical, KÖln, Germany.) was implanted in 16 patients (94%), and the other patient received Amplatzer PDA duct occluder device. All devices were implanted successfully in the defect. Post-device implantation, 2 patients (12%) developed bradyarrhythmia; one patient suffered from transient 2:1 heart block which resolved after 3 days with steroid therapy and the other developed asymptomatic sinus bradycardia which resolved spontaneously. None of the patients developed tachyarrhythmia during or after procedure.

Immediately post-procedure, residual shunt was detected in 11 patients (64.7%); 7 patients (63.6%) had mild, 3 patients (27.3%) had moderate, and 1 patient (9.1%) had large residual shunts. At 6-month follow-up, 4 patients (23.5%) had mild residual shunts. Significant valvular affection was present in 3 patients (17.6%) post-procedure; one patient developed mild aortic regurgitation, and 2 patients had mild tricuspid regurgitation.

### LV parameters at follow-up

Changes in echocardiographic LV parameters, LVEDD and LVESD Z-scores at 6-month follow-up are represented in Table 4.

**Table 4:** LV echocardiographic parameters before and after 6 months follow-up

Measurements	Before closure (median)	After 6 months (median)	*p* value
LVEDD/BSA (mm/m^2^)	46.5	42.9	0.03
LVESD/BSA (mm/m^2^)	31.7	26.7	0.02
LVEDV/BSA (ml/m^2^)	52.6	37.3	0.02
LVESV/BSA (ml/m^2^)	31.7	23.3	0.02
LV mass/BSA (gm/m^2^)	62.4	57.9	0.01
LVEDD Z-score	1.2	0.025	0.01
LVESD Z-score	1.7	− 0.35	0.04
EF (%)	65	65.5	0.96
BSA (m^2^)	0.82	0.9	0.002

BSA: Body surface area, EF: ejection fraction, LV: left ventricular, LVEDD: left ventricular end diastolic dimension, LVESD: left ventricular end systolic dimension, LVEDV: left ventricular end diastolic volume, LVESV: left ventricular end systolic volume

## Discussion

Our study demonstrated that percutaneous closure of VSD is associated with favorable LV remodeling demonstrated by significant reduction in indexed LV dimensions, volumes and mass. There was also significant reduction in LVEDD Z-score and LVESD Z-score.

VSD is one of the most common forms of congenital heart disease which may occur isolated or in combination with other congenital defects. Isolated large VSD, if left untreated, may be associated with unfavorable outcome due to chronic LV volume overload [10].

Due to pressure difference between systemic and pulmonary circulation, VSD is associated with left to right shunting of blood; this will lead to increased pulmonary blood flow and subsequently pulmonary venous return to the left sided heart chambers. This continued volume overload will eventually cause left side chambers dilatation and eccentric LV hypertrophy due to increased wall stress [3, 11].

The amount of left to right shunt associated with VSD is determined mainly by the defect size and the gradient between systemic and pulmonary circulation. In large VSD, the key determinant of left to right shunt amount is the relation between pulmonary and systemic vascular bed resistance; a relation that is variable and dependent on age. Due to the high pulmonary vascular resistance in neonatal period, the amount of shunt will be minimal in babies. With lung expansion and exposure of alveoli to oxygen, a potent pulmonary vasodilator, there will be fall in pulmonary vascular resistance and increase in left to right shunt and pulmonary blood flow [3, 12].

The chronic volume overload associated with VSD leads to structural changes at the myocyte level. The reversibility of such changes depends mainly on the duration and amount of volume overload. Thus, the age of VSD closure plays a pivotal role in the restoration of normal LV parameters post-volume overload removal [5].

Timely corrected VSD closure is associated with reduction in LV dimensions, volumes and masses; however, the time course of reduction in such parameters is not uniform with slower pace of LV mass regression compared to other parameters [6]. Such delay of LV mass regression is multifactorial; grade and duration of hypertrophy, structural changes associated with hypertrophy as myocardial atrophy and/or fibrosis and age of VSD correction [13].

For several decades, surgical closure was the treatment of choice for VSD with favorable effect on LV parameters. Pacileo et al. [14] demonstrated significant reduction in indexed LVEDV, LVESV and LV mass in patients who underwent surgical VSD closure after the age of 2 years old. Also, Jarmakani et al. [13] showed significant reduction in indexed LVEDV and LV mass. Furthermore, Jin cho et al. [11] showed significant reduction in indexed LVEDV at 3- and 12-months follow-up post-VSD closure. Similar to these studies, our study demonstrated significant reduction in indexed LV parameters which can be explained by change in patients BSA at follow-up due to continuous body growth during childhood period.

There is a controversy in the data determining the effect of VSD surgical closure on LV EF. Cordell et al. [5] showed that there was no significant difference between LV EF pre- and post-VSD closure and both were in the normal range. On the other hand, Jarmakani et al. [13] demonstrated significant reduction in EF post-VSD closure (60% vs. 55%, *p* < 0.02). This discrepancy in results may be related to the age of VSD correction, as delayed correction may be associated with irreversible changes at the myocyte level causing myocardial dysfunction [5, 6].

Due to abnormal loading conditions seen in patients with VSD in the form of increased LV preload due to left to right shunt and afterload reduction due ejection of blood into low resistance pulmonary circulation, any underlying myocardial dysfunction may be obscured [15]. Such abnormal loading conditions will lead to normal EF despite myocardial dysfunction, with VSD closure and correction of loading status; reduction in EF may occur.

Also, the surgical technique used and permanent damaging effect of intra-operative myocardial ischemia during intra-cardiac manipulation may have a deleterious effect on EF, a disadvantage that is not encountered by percutaneous closure as demonstrated by our study that showed no significant change in LV EF at follow-up [16].

Measurement of different cardiac structures by echocardiography plays a pivotal role in medical decision making in children with structural cardiac disease. Since body growth is a continuous process in this phase of life, normalization of echocardiographic dimensions to body size is a vital step for accurate analysis of these measurements [17]. For this purpose, calculation of Z score for the desired measurement is used. Similar to our study, Yang et al. [18] demonstrated significant reduction in LVEDD Z-score (*p* < 0.001) after percutaneous VSD closure and above all, there was no significant difference in LVEDD Z-score reduction between surgical and percutaneous VSD closure (*p* = 0.319). Also, Haddad et al. [19] reported reduction in LVEDD- Z score post-percutaneous VSD closure at the end of follow-up period.

The presence of significant residual shunt post-percutaneous VSD closure may be associated with unfavorable LV remodeling due to incomplete correction of volume overload status. The rate of residual shunt in our study is similar to what reported by the European VSD registry [9], but higher than what stated by another study (40%) [20]. In concordance to other studies [20, 21], our study showed improvement in the rate and the degree of residual shunt at follow-up which may be related to the coagulation effect of polyester fibers present in the device implanted in our patients [21].

## Limitations

First, the duration of follow-up was short and a longer period of follow-up is needed to confirm the regression of various LV parameters, maintenance of normal systolic function and absence of device related complications on the long term. Second, the number of procedures was low, and this was related to the fact that surgical closure is still the method of choice for VSD closure especially for symptomatic patients and thus, a larger number of patients are needed to confirm the findings of our study. Third, only one type of VSD occlude device was used in almost all patients and thus, the results of our study especially procedural success and complications rate cannot be generalized to other devices.

## Conclusions

Percutaneous VSD closure is associated with favorable LV remodeling as demonstrated by improvement of indexed LV volumes, dimensions and mass and preservation of normal LV systolic function. The results of our study need to be validated in larger study population.

## Data Availability

The datasets used and/or analyzed during the current study are available from the corresponding author on reasonable request.

## Abbreviations

AV: Aortic valve
BMI: Body mass index
BSA: Body surface area
ECG: Electrocardiogram
EF: Ejection fraction.
IQR: Interquartile range
IVS: Interventricular septum
LA: Left atrium
LV: Left ventricular
LVEDD: Left ventricular end-diastolic dimension
LVESD: Left ventricular end-systolic dimension
LVEDV: Left ventricular end-diastolic volume
LVESV: Left ventricular end-systolic volume
Qp/Qs: Ratio of pulmonary blood flow to systemic blood flow
RV: Right ventricle
VSD: Ventricular septal defect
